# A Standardized Unpredictable Chronic Stress Protocol for Adult Zebrafish

**DOI:** 10.1002/cpz1.70376

**Published:** 2026-04-24

**Authors:** Matheus Gallas‐Lopes, Ana P. Herrmann, Angelo Piato

**Affiliations:** ^1^ Laboratório de Neurobiologia e Psicofarmacologia Experimental (PsychoLab), Departamento de Farmacologia ICBS, UFRGS Brazil; ^2^ Laboratório de Psicofarmacologia e Comportamento (LAPCOM), Departamento de Farmacologia ICBS, UFRGS Brazil; ^3^ Programa de Pós‐graduação em Farmacologia e Terapêutica ICBS, UFRGS Brazil

**Keywords:** anxiety, stress‐induced alterations, stress‐related disorders, unpredictable chronic stress, zebrafish

## Abstract

The unpredictable chronic stress (UCS) protocol, originating in the 1980s, has contributed to depression research by inducing behavioral and physiological changes in rodents that resemble the symptoms observed in patients. Its translational potential led to widespread adoption, but adaptations and variations to the protocol have raised concerns about reproducibility. Over a decade ago, our laboratory adapted the UCS protocol for zebrafish (*Danio rerio*), aiming to bridge species‐specific gaps in stress research. Since then, several studies have reported alterations in outcomes related to the clinical manifestations of stress‐related disorders, providing evidence on the applicability of the model for testing drugs and other therapeutic strategies. However, there is substantial heterogeneity in protocols and research findings, hindering comparability across studies. To address these challenges, we describe a 14‐day UCS protocol for adult zebrafish in detail, involving a series of varied stressors administered twice daily. This comprehensive protocol includes stressors, such as tank changes, net chasing, overcrowding, low water levels, and temperature fluctuations. Each step is designed to ensure reproducibility and to mimic the unpredictable and varied nature of stress experienced by humans. By establishing standardized procedures, we aim to enhance the consistency, reliability, and translational potential of UCS research using zebrafish, ultimately contributing to more robust and comparable findings across studies. © 2026 The Author(s). Current Protocols published by Wiley Periodicals LLC.

**Basic Protocol**: UCS protocol for adult zebrafish

## INTRODUCTION

The unpredictable chronic stress (UCS) protocol has its origins in the early 1980s, with the publication of a series of studies by Katz and collaborators (Katz, 1982; Katz & Hersh, 1981; Katz et al., 1981). In these studies, rodents were exposed sequentially to a series of mild to severe stressors with the purpose of inducing behavioral and physiological alterations relevant to the study of mood and anxiety disorders.

Apart from modeling anhedonia, the UCS protocol also elicits anxiety‐like behaviors, cognitive impairments, hormonal and neurochemical imbalances, weight loss, and other alterations reminiscent of those observed in individuals with major depressive disorder, thereby establishing both face and construct validity to the model (Willner, 1997; Willner et al., 1987). This parallels the predictive validity demonstrated by rodents' response to treatments within 3 to 4 weeks, reflecting the time course of antidepressant effectiveness in clinical settings. As a result, the protocol has seen widespread adoption across laboratories, emerging as a tool for exploring the neurobiological mechanisms underlying stress‐related disorders and the effects of novel drug candidates (Willner, 1997, 2017).

Over the years, there have been several adaptations to the original UCS protocol, including modifications in duration, types of stressors, and even the choice of species. Notably, an adaptation was made for studies involving zebrafish (*Danio rerio* Hamilton, 1822) (Piato et al., 2011), an emerging model organism in neuroscience at the time. Cross‐species approaches increase the external validity of preclinical studies aimed at assessing the efficacy of therapeutic interventions (Barron et al., 2020; Berg et al., 2024). Thus, adapting the UCS protocol for zebrafish is of relevance to reduce species‐specific biases inherent to studies predominantly conducted with rodents, and ultimately potentiate translational success.

### Development of the Protocol

The efforts to adapt the UCS protocol for zebrafish research started just over a decade ago, led by Piato et al. (2011). At that point, an extensive behavioral repertoire had been described for zebrafish. However, behavioral research using zebrafish remained in its early stages as compared to rodents, with the majority of studies concentrating on natural and innate behaviors (Buske & Gerlai, 2011; Gerlai, 2010a, 2010b; Serra et al., 1999). Although there was evidence suggesting zebrafish might be suitable for studying physiological and biochemical processes that are conserved between species and of relevance to human pathologies, the assessment of the behavioral response to stress in this model organism had been scarcely characterized (Champagne et al., 2010; Egan et al., 2009).

One of the main reasons to adapt the UCS protocol for zebrafish is that their stress response is regulated by a system similar to the mammalian hypothalamus–pituitary–adrenal (HPA) axis (Gorelick & Habenicht, 2020). Stress responses in zebrafish are mediated by the hypothalamus–pituitary–interrenal (HPI) axis, which regulates cortisol release in response to environmental challenges (de Abreu et al., 2021; Gorelick & Habenicht, 2020). Acute exposure to stressors like overcrowding (Ramsay et al., 2006), net handling (Ramsay et al., 2009), or predator encounters (Barcellos et al., 2007), stimulate the HPI axis, triggering an increase in cortisol levels and inducing behavioral changes in adult zebrafish.

The UCS protocol involves exposing animals to a series of stressors sequentially and in an unpredictable way to consistently provoke significant and lasting behavioral and physiological responses (Atrooz et al., 2021; I. Tran & Gellner, 2023). The adaptation of the UCS protocol from rodents to zebrafish involved incorporating stressors known to affect fish, drawing from both zebrafish research and insights from fish farming practices (Barcellos et al., 2007; Demers & Bayne, 1997; Ramsay et al., 2006, 2009; Rotllant & Tort, 1997; Zuccarelli & Ingermann, 2007). The selected stressors were designed to suit the aquatic environment, aligning with the natural habitat of zebrafish and addressing the distinct physiological requirements of this species.

The first study to adapt the UCS protocol for zebrafish used two different stress protocol durations (7 or 14 days), with fish subjected twice daily to one of the following stressors: restraint stress, heating tank water to 33°C, social isolation, cooling tank water to 23°C, crowding of 10 animals in a beaker, exposure to a predator (*Amatitlania nigrofasciata* Günther, 1867), lowering water levels in tanks, tank water replacement, tank change, and chasing with a net. Exposure to UCS for 7 or 14 days induced behavioral, endocrine, and molecular alterations, including anxiety‐like behavior, impaired memory performance, and increased cortisol levels, supporting the validity of the model (Piato et al., 2011).

Following the publication of this pioneering study, our group has successfully applied the UCS protocol across several experimental contexts. Researchers within our laboratory later replicated the earlier results showing that UCS induces behavioral and neuroendocrine alterations in zebrafish. They further demonstrated that anxiolytic and antidepressant drugs can prevent these effects, adding predictive validity to the model (Marcon et al., 2016). This suggests that the UCS protocol could potentially be used as a screening tool for evaluating new drugs aimed at treating psychiatric disorders with stress‐related origins. In subsequent studies, the protocol was also applied to investigate non‐pharmacological interventions such as environmental enrichment (EE) (Marcon, Mocelin, Benvenutti, et al., 2018; Marcon, Mocelin, Sachett, et al., 2018). EE mitigated the effects induced by UCS on behavior and cortisol levels and prevented the impacts on oxidative stress parameters and pro‐inflammatory markers. Furthermore, the UCS protocol has been employed to test various molecules with antioxidant, anti‐inflammatory, neuroprotective, anxiolytic, and antidepressant properties (Bertelli et al., 2021; Marcon et al., 2019; Mocelin et al., 2019; Sachett et al., 2022).

The UCS protocol proposed for zebrafish has been adapted by multiple laboratories to accommodate different research questions and infrastructure. This flexibility has contributed to variability in stressor selection, duration, and outcome measures across studies. To better understand the overall consistency and validity of the model, we conducted a systematic review and meta‐analysis covering the first decade of UCS use in zebrafish research (Gallas‐Lopes, Bastos, et al., 2023). The goal was to evaluate the strength of evidence supporting UCS‐induced behavioral and biochemical alterations relevant to stress‐related disorders and to identify potential sources of heterogeneity.

Overall, the meta‐analysis demonstrated that UCS exposure consistently increases anxiety‐ and fear‐related behaviors and elevates cortisol levels compared to control animals, while locomotor activity is generally reduced. Effects on social behavior were less consistent across studies, highlighting areas requiring further investigation. Importantly, subgroup analyses indicated that protocol duration plays a critical role in determining outcomes, with stress regimens >7 days producing more robust behavioral and physiological effects.

The systematic review also revealed considerable variability in methodological and reporting practices across studies, including inconsistent reporting of randomization, blinding, and sample size estimation. Such variability may contribute to heterogeneity in reported outcomes and complicate comparisons between studies. These findings reinforce the need for clear and standardized procedures when implementing UCS in zebrafish.

Taken together, the available evidence supports the use of a 14‐day UCS protocol as a practical balance between experimental feasibility and the induction of measurable stress‐related behavioral and biochemical alterations (Gallas‐Lopes, Bastos, et al., 2023). By providing a standardized framework based on accumulated experimental experience and published literature, this article aims to improve reproducibility and facilitate comparisons across laboratories.

### Applications of the Method

The UCS protocol has been instrumental in exploring the neurobiology of stress and evaluating the effects of pharmacological and non‐pharmacological interventions on behavioral and biochemical responses to stress. Researchers have used this protocol to test the potential anxiolytic and anti‐stress effects of drug candidates, demonstrating its efficacy in mitigating stress‐induced changes (Benneh et al., 2017; Bertelli et al., 2021; Costa de Melo et al., 2019; Dos Santos Sampaio et al., 2018; Marcon et al., 2019; Mocelin et al., 2019; R. G. Reddy et al., 2018, 2019; Sachett et al., 2022). Additionally, it has been validated with commercially available psychotropic medications, allowing for comparisons between new drugs and established treatments (Marcon et al., 2016).

Beyond pharmacological testing, the UCS protocol has facilitated studies on the neurobiology of stress, examining behavioral, physiological, and molecular outcomes (Chakravarty et al., 2013; Demin et al., 2020; Fulcher et al., 2017; Grzelak et al., 2017; Manuel et al., 2014; Pavlidis et al., 2015; B. R. Reddy et al., 2021). Furthermore, the protocol has been used to evaluate non‐pharmacological interventions such as EE (Marcon, Mocelin, Benvenutti, et al., 2018; Marcon, Mocelin, Sachett, et al., 2018). This adaptability makes the UCS protocol a tool for studying stress‐related conditions like anxiety, depression, post‐traumatic stress disorder (PTSD), and increased susceptibility to substance abuse, providing insights into potential therapeutic strategies (Al‐Zoubi et al., 2024; Gallas‐Lopes, Bastos, et al., 2023; Nguyen et al., 2014).

### Comparison With Other Methods

Alternative approaches to study stress in zebrafish include acute stress paradigms, repeated exposure to a single stressor, and genetic or pharmacological models that target stress‐related pathways. Acute stress protocols typically induce transient behavioral and physiological alterations, whereas repeated exposure to the same stressor may lead to habituation and reduced stress responsiveness over time (Atrooz et al., 2021; Kirsten et al., 2021; Quadros et al., 2021). In contrast, the UCS protocol combines multiple stressors applied in an unpredictable sequence, which helps sustain behavioral and endocrine alterations relevant to stress‐related disorders.

Other models, such as chronic mild stress (CMS) or prolonged unpredictable stress protocols, share conceptual similarities with UCS but often vary in duration, stressor selection, and implementation (Demin et al., 2020; Fulcher et al., 2017; Song et al., 2018). While longer protocols have been described, extended durations may increase logistical complexity without necessarily improving outcome detection. Based on available evidence and our previous work, a 14‐day UCS regimen provides a practical balance between experimental feasibility and induction of measurable behavioral and biochemical alterations (Gallas‐Lopes, Bastos, et al., 2023).

Although the UCS model in zebrafish reproduces several behavioral, endocrine, and molecular alterations associated with chronic stress, some limitations should be acknowledged. In particular, zebrafish lack a direct equivalent of rodent anhedonia paradigms, and the predictive validity of the model for major depressive disorder remains less extensively characterized (de Abreu et al., 2018, 2022; Marx et al., 2023). Despite these limitations, the model provides a useful framework for investigating mechanisms and potential interventions related to stress‐related phenotypes.

This article describes a 14‐day UCS regimen for adult zebrafish administered twice daily. The Basic Protocol incorporates diverse stressors (e.g., tank changes, net chasing, overcrowding, low water level, and temperature fluctuations) to preserve unpredictability and support reproducibility across laboratories. By standardizing key experimental conditions, stressor application, and optional downstream behavioral and biochemical assessments, this protocol aims to reduce discrepancies in the zebrafish UCS literature and facilitate reliable, comparable outcomes across studies.

## STRATEGIC PLANNING

### Experimental Design

#### Subjects and housing

This protocol is designed for adult zebrafish (*Danio rerio*), typically ≥90 days post‐fertilization. Animals should be acclimated to laboratory conditions for ≥1 week prior to experimentation and monitored to ensure good health status. Zebrafish should be maintained under standard housing conditions, including controlled temperature (27° ± 1°C), stable water quality, and a consistent light/dark cycle (e.g., 14/10 hr). Housing density should follow established guidelines to maintain welfare and minimize baseline stress (Aleström et al., 2020; Kütter et al., 2023).

#### Environmental enrichment

Most UCS experiments have been conducted using “barren” tanks, which lack any form of EE. Studies have shown that enriched environments may reduce anxiety‐like behavior and improve welfare in zebrafish (Gallas‐Lopes, Benvenutti et al., 2023). In our experience, EE can attenuate behavioral, endocrine, and oxidative stress alterations induced by UCS (Marcon, Mocelin, Benvenutti, et al., 2018; Marcon, Mocelin, Sachett, et al., 2018). Therefore, when enrichment is used, mild approaches (e.g., PVC pipes or live food such as *Artemia salina*) are recommended to maintain animal welfare while preserving sensitivity to stress‐induced effects.

#### Allocation to experimental groups

To ensure the validity and reliability of the experimental results, zebrafish should be allocated to treatment groups using a systematic and randomized approach. Each experimental group should originate from at least two home tanks to avoid tank effects, which could introduce bias due to differences in tank environments. The allocation process should follow block randomization procedures to ensure that variables, e.g., sex, weight, age (if different age groups are involved), and the two different home tanks are balanced across the experimental groups. Although it is challenging due to the lack of clear sexual dimorphism in zebrafish, researchers should make efforts to balance the sex ratio within experimental groups. To ensure accurate sex determination, it is recommended to perform gonadal dissection after euthanasia. This method helps to counterbalance potential confounding factors and minimize bias. Randomness in the allocation process can be achieved using a random number table or a computer random number generator (e.g., https://www.random.org). It is essential to avoid non‐random allocation methods, including allocation based on investigator judgment or preference, results from laboratory tests, or availability of the intervention, as these can introduce bias and compromise the integrity of the experiment.

#### Sample size

To avoid running an underpowered experiment, the sample size should be calculated *a priori* based on the primary outcome and effect size of interest, taking into consideration the experimental design and planned statistical analysis. For measures, such as social behavior, anxiety, locomotion, and cortisol, the summary effect sizes (or an individual effect size if a specific study is deemed more appropriate) reported in our meta‐analysis can provide guidance on what is reasonable to expect. For example, in studies assessing anxiety/fear behavior, an effect size of 1.09 can be investigated with a power of 0.95 and an alpha of 0.05 with a minimum of 23 animals per experimental group in a two‐group design. To account for inflated effect sizes, larger sample sizes should be used.

#### Housing during the UCS protocol

During the UCS protocol, housing conditions should remain consistent with standard husbandry to minimize unintended stress. Tanks from different experimental groups should be maintained in the same room and randomly distributed within the housing system to avoid environmental bias. Visual barriers should be installed on the sides of tanks to prevent behavioral interference between groups; however, the front of the tank should remain unobstructed to allow animals to acclimate to experimenters and routine husbandry procedures. Housing tanks should be coded, when possible, to reduce caregiver awareness of group allocation and minimize potential bias. To reduce handling‐related stress, a net should remain inside each tank throughout the experiment. Recirculating water systems should be avoided, as stressed fish may release alarm substances that could affect animals housed in connected tanks; static tanks with individual filtration are recommended. After allocation to experimental groups, the UCS protocol should begin the following day. Tank cleaning and other disturbances should be minimized during the protocol, and feeding routines should remain consistent across groups and days.

#### Experimental groups

At a minimum, an unstressed control group and a UCS group are needed. If additional interventions are included, such as drugs or non‐pharmacological interventions, an additional UCS group receiving the intervention is necessary. If the intervention is administered using a vehicle solution, and its potential effects are unclear, a pilot study should be conducted to evaluate the effects of the vehicle before proceeding with the UCS protocol.

Although procedural equivalence and blinding for the UCS protocol are impossible at the level of the experimenter implementing the protocol, all other interventions should strive for equivalence and blinding. For example, if the UCS group is taken from the housing tank to receive a pharmacological intervention, the control group should also undergo the same procedure, being exposed to a vehicle. Additionally, where possible, use codes to blind the person administering the treatment to avoid bias.

#### Drug treatments (optional)

In experiments designed to understand the effects of drugs on zebrafish, it is essential to choose an appropriate administration route. The two most common methods for drug delivery in zebrafish are immersion and intraperitoneal injection. The specific steps for each route are well‐documented in several research papers and protocols (Cachat et al., 2010; Sachett et al., 2022). Researchers should refer to these sources to ensure accurate and effective administration. Proper controls, randomization, and blinding should be incorporated to minimize bias and validate the experimental results.

#### Stressors used

**Figure 1:**
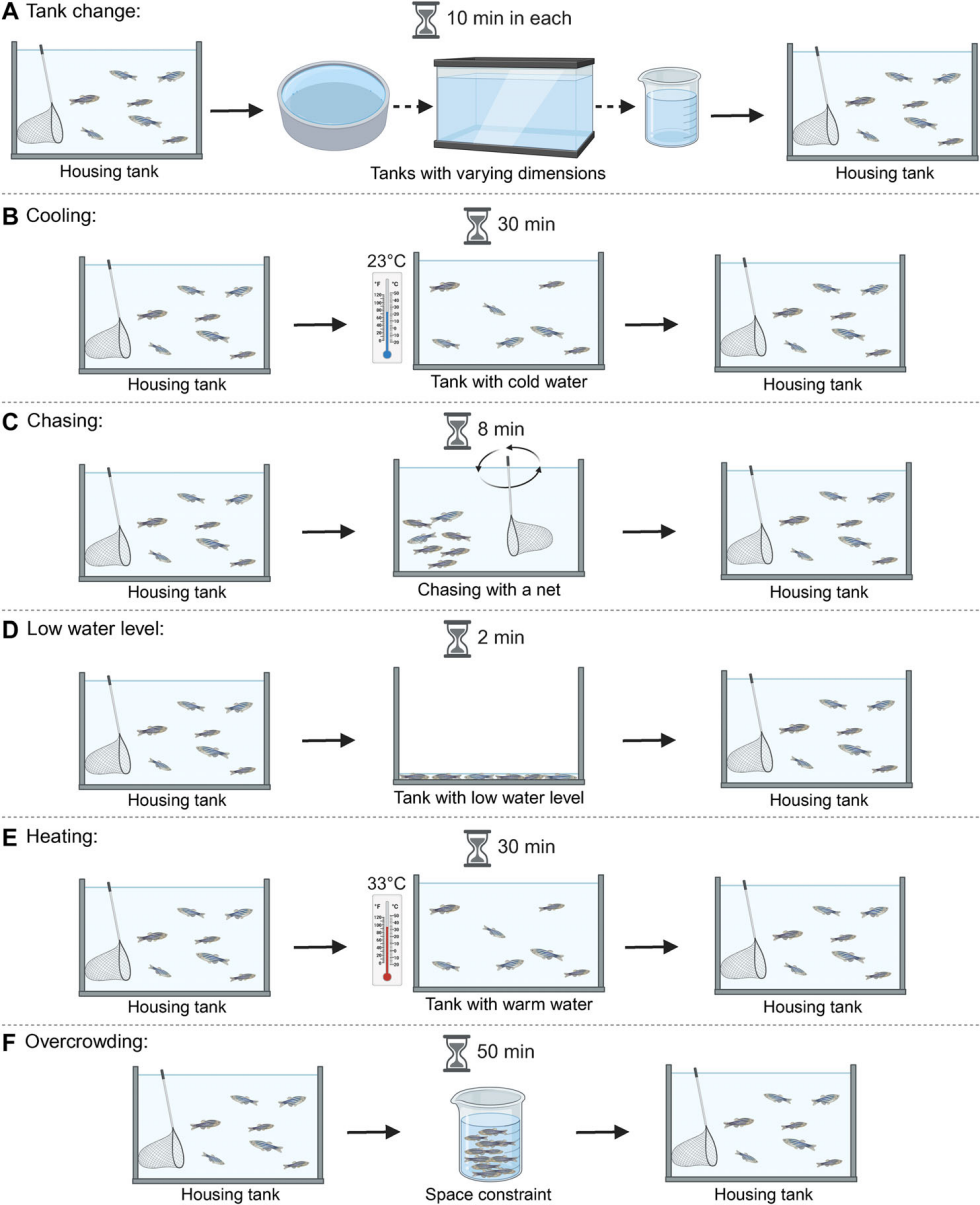
Illustration of stressors: (**A**) Tank change, (**B**) Cooling, (**C**) Chasing, (**D**) Low water level, (**E**) Heating, (**F**) Overcrowding. Stressors are applied randomly and twice daily for 14 days to induce stress in zebrafish. Created with BioRender.

As mentioned previously, while several stressors have been tested, we detail below and in Figure 1 the ones that have been extensively tested and proven effective in inducing a stressed phenotype in zebrafish within our research group. Minor adjustments to these stressors should not significantly impact the UCS outcomes. The stressors are applied twice daily for 14 days at varied times to prevent habituation and maintain unpredictability.

Tank change: Fish from the same housing tank are relocated to new tanks with varying dimensions, where they remain for 10 min per transfer. This process is repeated three times consecutively, introducing environmental changes and disturbances to the fish habitat.
Cooling: Fish are transferred to a tank where the water temperature is set to 23°C and maintained for 30 min. This rapid temperature reduction serves as a thermal stressor.
Chasing: Fish are actively pursued with a net for 8 min, either within the housing tank or in a designated area. This chasing procedure induces acute physical stress, mimicking predator evasion responses.
Low water level: Fish are placed in a tank with reduced water levels, exposing their dorsal body parts for 2 min. This exposure to low water levels replicates stressful environmental conditions.
Heating: Fish are transferred to a tank where the water temperature is set to 33°C and maintained for 30 min. This elevated temperature serves as a thermal stressor.
Overcrowding: Fish are confined in a beaker/tank with a density of 45 fish/L for 50 min (e.g., 9 fish in 200 ml water). This overcrowding condition induces social stress and confinement, reflecting challenges in social interactions and space constraints.

Throughout these procedures, the water in the tanks is maintained at appropriate physical, chemical, and biological conditions, except during the heating and cooling stressors, where the temperature is intentionally altered. The control group will receive standard care for 14 days to serve as a baseline comparison and should remain undisturbed during this period, except for the periodic movement of the net inside the tank every other day to ensure habituation and prevent an acute stress response during testing. It is crucial that the handling of animals from the housing tank to the stressors and back is performed as swiftly as possible to prevent hypoxia and minimize additional stress. This handling should be conducted by trained personnel to ensure the welfare of the animals and to avoid unnecessary distress.

#### Alternative stressors

While the stressors mentioned above are the easiest to apply across different laboratories and have been consistently tested in our lab over the years, alternative stressors can also be used effectively. For examples and detailed descriptions of additional stressors, please refer to our systematic review (Gallas‐Lopes, Bastos, et al., 2023).

#### Stressors schedule

**Figure 2:**
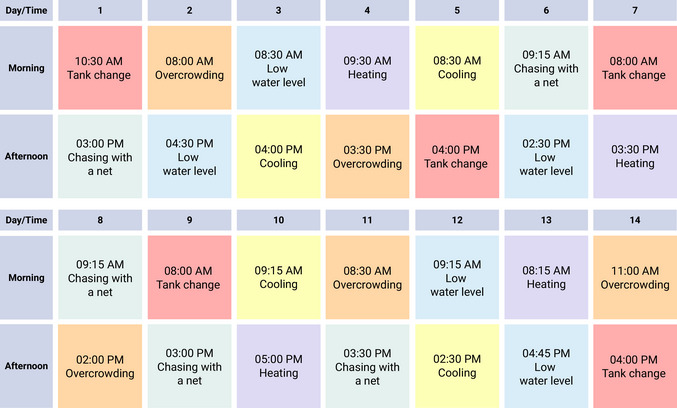
Example of a randomized schedule of stressors across the 14‐day UCS protocol. The figure illustrates a representative randomization of stressors distributed across days and time periods (morning and afternoon sessions). This schedule can be used directly or adapted by researchers implementing the protocol, helping to ensure balanced presentation of stressors while maintaining unpredictability. Created with BioRender.

The stressors schedule is a critical component to ensure the effectiveness and reliability of the UCS protocol. Stressors should be administered randomly twice daily for a total of 14 days to prevent habituation and maintain the unpredictability of the protocol. Each day, one stressor should be presented in the morning between 8:00 a.m. and 11:00 a.m., and another in the afternoon between 1:00 p.m. and 5:00 p.m. To achieve randomness in the presentation, refer to the table provided in Figure 2 or use a random number generator, (e.g., https://www.random.org), while ensuring that the schedule is balanced to avoid repeatedly concentrating the same stressor at the same time of day. It is essential to account for the number of presentations of each stressor and to randomize the hours of presentation in both the morning and afternoon sessions.

#### Behavioral assessments and biochemical/molecular assessments (optional)

Following completion of the UCS protocol, behavioral and/or biochemical outcomes may be assessed according to the objectives of the study. Common behavioral assays sensitive to anxiety‐like responses include the novel tank test, light/dark test, open tank test, and social preference test (Cachat et al., 2010; Maximino et al., 2010; Kysil et al., 2017; S. Tran & Gerlai, 2016; Bertelli et al., 2021; Sachett et al., 2022; Gallas‐Lopes, Bastos, et al., 2023; Neves et al., 2026). Frequently used biochemical or molecular endpoints include whole‐body cortisol levels, oxidative stress markers, and gene expression related to stress pathways (Marcon, Mocelin, Sachett, et al., 2018; Sachett et al., 2022; Gallas‐Lopes, Bastos, et al., 2023). To avoid confounding acute stress effects, assessments should be performed on the day following the last stress session.

#### Animal welfare

Fish should be monitored daily for any signs of stress or distress, such as abnormal behavior or physical injuries. Humane endpoints should be established to guide the early euthanasia of fish exhibiting severe or persistent stress symptoms that do not resolve or if they are unable to recover, thus minimizing suffering. The experimental protocol should adhere to ethical guidelines and obtain approval from the relevant ethics committee to ensure compliance with all animal welfare regulations.

#### Euthanasia and sex confirmation

Zebrafish should be euthanized according to established ethical guidelines (e.g., AVMA Guidelines for the Euthanasia of Animals) using accepted methods, such as MS‐222 overdose or rapid chilling (Underwood & Anthony, 2020). Because zebrafish do not exhibit clear external sexual dimorphism, gonadal dissection after euthanasia is recommended to confirm sex when relevant to the experimental design.

#### Good research and report practices

To ensure the quality and integrity of research involving animal experiments, researchers should follow established guidelines and practices. Begin by consulting SYRCLE's Risk of Bias Tool to identify and address potential sources of bias in your experimental design (Hooijmans et al., 2014). Adhere to PREPARE guidelines to plan and execute animal research with attention to ethical considerations and procedural rigor (Smith et al., 2018). Use ARRIVE guidelines to improve the transparency and completeness of your reporting (Percie du Sert et al., 2020). Preregister your experiments, hypotheses, and methods to enhance the credibility of your research and avoid questionable practices. Finally, make your raw data accessible in open repositories like the Open Science Framework (https://osf.io/) to promote transparency and facilitate reproducibility.

*NOTE*: All protocols involving animals must be reviewed and approved by the appropriate Animal Care and Use Committee and must follow regulations for the care and use of laboratory animals.

## UNPREDICTABLE CHRONIC STRESS PROTOCOL FOR ADULT ZEBRAFISH

This protocol describes a 14‐day unpredictable chronic stress (UCS) regimen for adult zebrafish, in which animals are exposed twice daily to a randomized sequence of physical and environmental stressors. Stressors are applied in an unpredictable order and at variable times to prevent habituation and to induce sustained behavioral and physiological stress responses. This protocol produces stress‐related alterations, including increased anxiety‐like behavior and elevated cortisol levels, providing a robust model for studying chronic stress and testing pharmacological or non‐pharmacological interventions in adult zebrafish.

### Materials

Adult zebrafish (*Danio rerio*)
Reagents for biochemical/molecular analysis (optional)
Ethyl 3‐aminobenzoate methanesulfonate salt (MS‐222) (Sigma‐Aldrich, cat. no. A5040), if needed for euthanasia
Chilled water (if needed for euthanasia)

G*Power (https://www.psychologie.hhu.de/arbeitsgruppen/allgemeine‐psychologie‐und‐arbeitspsychologie/gpower)
Fish tank(s) (static or within circulating aquatics system)
Filtration systems (e.g., filters, pumps)
Heating units (e.g., aquarium heaters)
Aeration units
PVC pipes for enrichment
Net for each aquarium
Lighting systems (e.g., light/dark cycle timers)
Stopwatch Water testing kits (e.g., pH meter, ammonia, chlorine test kits)
Labels to identify aquariums
Permanent markers
Additional tanks for the stress protocol
Nets for chasing
Beakers or containers for overcrowding
Equipment for tank changes (at least three tanks of varying sizes and shapes)
Thermometer
Equipment to cool water (e.g., ice machine)
Equipment to heat water
Setup for behavioral analysis (optional)
Beakers for euthanasia
Dissection tools (e.g., scissors, forceps, scalpel, tweezers)

*CAUTION*: When manipulating MS‐222 (tricaine), wear protective gloves, clothing, eye, and face protection.

The following step‐by‐step procedure is based on the example of randomized stressors presented in Figure 2. As the protocol inherently involves stressing the animals and intentionally disrupting their welfare, it is crucial to ensure the highest level of experimental rigor to avoid waste and ensure reproducibility.

#### Sample size estimation

1 Identify the primary outcome measure for your experiment (e.g., anxiety‐like behavior, cortisol levels). Use existing literature or preliminary data to estimate the expected effect size for this outcome measure or set the smallest effect size of interest. If unsure, refer to effect sizes reported in meta‐analyses or similar studies.
2 Based on the expected effect size, desired power (typically 0.90), and significance level (alpha, typically 0.05), calculate the required sample size using an appropriate tool such as G*Power.

#### Prepare housing tanks (4 tanks)

3 Set up standard aquaria with filtration systems to maintain water quality, heater, aeration, tank lids, and PVC pipes for enrichment. Choose the adequate tank size to have groups with a density of ∼5 individuals/L.
  A wrong tank size can affect fish well‐being and experimental outcomes.
4 Place a net inside each aquarium. Ensure the net does not obstruct the animal's movement or interfere with their normal activities. The continuous presence of a net inside the aquarium during the experiment will facilitate the manipulation of the animals for behavioral testing. Remember to move the net inside the control group's aquarium every other day to acclimate the animals to handling.
5 Fill the housing tanks with water following optimal characteristics for zebrafish. Check temperature daily, and monitor pH, conductivity, chlorine, and ammonia levels routinely to ensure they remain within optimal ranges for zebrafish health.
  Deviations can lead to inaccurate results or health issues for the fish.
6 Install visual barriers on tank sides to prevent fish from observing other groups. Keep the front of tanks unobstructed to allow fish to acclimate to experimenters and caregivers.
7 Use codes to label tanks, ensuring caregivers are blind to the experimental groups. House fish from different experimental groups in the same room, with random housing assignments to avoid bias.
8 Randomly allocate fish to the different treatment groups. Use block randomization to balance variables, such as sex, weight, and age across the treatment groups.
9 Transfer the allocated zebrafish to each tank. Handle the fish gently and swiftly to minimize stress during the transfer process.

##### Pause point

This pause point is to prepare to start the stress protocol the next day. This pause ensures that all necessary preparations are in place and that housing conditions are fully optimized before initiating the stress protocol. Starting the protocol the following day allows for a thorough final check, reducing the risk of issues and ensuring the experiment proceeds under optimal conditions.

#### Stress session 1 (tank change)

10 Choose three tanks of varying shapes and sizes to introduce environmental changes. Ensure that each tank is clean and free from residues.
11 Fill each tank with water matching the optimal conditions for zebrafish. Ensure that the water level is appropriate for the fish.
12 Gently net the fish from the original tank and transfer them to the new tank. Ensure that the transfer is as swift as possible to minimize additional stress. Use a lid or a similar cover to prevent fish from jumping out the tank.
13 Allow the fish to remain in the new tank for 10 min. Ensure the second tank is ready and filled with water at the appropriate temperature.
14 Gently move the fish from the first tank to the second tank. Use a net or a suitable device to avoid causing undue stress or injury to the fish. Use a lid or a similar cover to prevent fish from jumping out the tank.
15 Allow the fish to remain in the new tanks for 10 min. Ensure the third tank is ready and filled with water at the appropriate temperature.
16 Gently move the fish from the second tank to the third tank using a net or another suitable method to minimize stress. Use a lid or a similar cover to prevent fish from jumping out the tank.
17 Allow the fish to remain in the new tanks for 10 min.
18 Gently move the fish from the third tank back to the home tank using a net or another suitable method to minimize stress. Use a lid or a similar cover to prevent fish from jumping out the home tank.
19 Observe the fish as they acclimate to ensure they settle back into their home environment without undue stress.

##### Pause point

Leave the fish undisturbed in their home tank until the next stress session, as determined by the randomized schedule.

#### Stress session 2 (chasing with a net)

20 Ensure that the home tank is equipped with the net that will be used for the chasing session.
21 Actively chase the fish within the home tank using the net for 8 min. Ensure that the metal or plastic frame of the net does not make direct contact with the fish to prevent injuries that could compromise their health.
22 After 8 min, stop chasing and leave the net inside the tank.
23 Observe the fish as they acclimate to ensure they settle back into their home environment without undue stress.

##### Pause point

Leave the fish undisturbed in their home tank until the next stress session, as determined by the randomized schedule.

#### Stress session 3 (overcrowding)

24 Select a beaker or small tank suitable for overcrowding. Ensure that the beaker/tank is clean and free from residues.
25 Fill each beaker/tank with water matching the optimal conditions for zebrafish.
26 Gently transfer the fish from their home tank into the overcrowding beaker/tank.
27 Place the fish into the beaker/tank at a density of 45 fish/L.
28 Keep the fish in the overcrowded beaker or tank for 50 min.
29 After 50 min, gently transfer the fish back to their home tank. Use a lid or a similar cover to prevent fish from jumping out the home tank.
30 Observe the fish as they acclimate to ensure they settle back into their home environment without undue stress.

##### Pause point

Leave the fish undisturbed in their home tank until the next stress session, as determined by the randomized schedule.

#### Stress session 4 (low water level)

31 Select an appropriate tank for the low water level stressor. Select a larger tank to accommodate the low water level, ensuring the fish have sufficient space to disperse even with the reduced water volume. Ensure that each tank is clean and free from residues.
32 Gently transfer the fish from their home tank into the prepared low water level tank. Gradually lower the water level in the tank to expose the dorsal body parts of the fish. The water level should be sufficient to fully submerge the fish's heads to prevent severe hypoxia.
33 Expose the fish to the low water level for 2 min.
34 After 2 min, gently transfer the fish back to their home tank. Use a lid or a similar cover to prevent fish from jumping out the home tank.
35 Observe the fish as they acclimate to ensure they settle back into their home environment without undue stress.

##### Pause point

Leave the fish undisturbed in their home tank until the next stress session, as determined by the randomized schedule.

#### Stress session 5 (low water level)

36 See steps 31 to 35.

##### Pause point

Leave the fish undisturbed in their home tank until the next stress session, as determined by the randomized schedule.

#### Stress session 6 (cooling)

37 Select and prepare a tank for the cooling stress session, ensuring it is clean and filled with water.
38 Adjust the water temperature to 23°C.
39 Gently transfer the fish from their home tank to the tank with cold water.
40 Leave the fish in the tank with cold water for 30 min. Monitor the water temperature to maintain it at 23°C throughout the session. Use an ice bath or reduce the temperature in the room if possible.
41 After 30 min, gently return the fish to their home tank. Use a lid or a similar cover to prevent fish from jumping out the home tank.
42 Observe the fish as they acclimate to ensure they settle back into their home environment without undue stress.

##### Pause point

Leave the fish undisturbed in their home tank until the next stress session, as determined by the randomized schedule.

#### Stress session 7 (heating)

43 Select and prepare a tank for the heating stress session, ensuring it is clean and filled with water at the appropriate level.
44 Adjust the water temperature to 33°C.
45 Gently transfer the fish from their home tank to the tank with warm water.
46 Leave the fish in the tank with warm water for 30 min. Monitor the water temperature to maintain it at 33°C throughout the session. Use a heater with a thermostat, a kettle to heat water, or other temperature control methods.
47 After 30 min, gently return the fish to their home tank. Use a lid or a similar cover to prevent fish from jumping out the home tank.
48 Observe the fish as they acclimate to ensure they settle back into their home environment without undue stress.

##### Pause point

Leave the fish undisturbed in their home tank until the next stress session, as determined by the randomized schedule.

#### Stress sessions 8 to 28

49 Continue applying stressors according to the randomized schedule until completion of the 14‐day protocol (two stress sessions per day). For each session, follow the corresponding procedure described above for the selected stressor (tank change, chasing, overcrowding, low water level, cooling, or heating).

##### Pause point

Leave the fish undisturbed in their home tank until the next stress session, as determined by the randomized schedule.

#### Behavioral assessments (optional)

50 Perform the behavioral assessments of choice.

#### Euthanasia and collection of samples

51 At the end of the protocol, euthanize zebrafish according to established guidelines, followed by tissue collection and storage under appropriate conditions for downstream analyses. Gonads should be dissected to confirm sex. Disposal of carcasses and solutions should follow institutional and regulatory guidelines.

#### Biochemical/molecular assessments (optional)

52 Perform the biochemical/molecular assessments of choice.

## COMMENTARY

### Troubleshooting

Common problems that may be encountered during the UCS procedure, along with possible causes and recommended solutions, are summarized in Table 1.

**Table 1:** Troubleshooting Guide for the UCS Protocol in Adult Zebrafish

Problem	Possible cause	Solution
Step 5: Addressing deviations in water quality parameters	Heater/thermostat failure; inadequate filtration; overfeeding; insufficient water changes; water source not conditioned/dechlorinated; poor calibration of meters/kits	If the water temperature is not within the ideal range, adjust the heating units accordingly and regularly monitor the temperature to maintain stability; if the pH is outside the desired range, use pH regulators to bring it back to the ideal levels; if chlorine is detected in the water, use a chlorine neutralizer to eliminate it and ensure that the water source is free from chlorine or use dechlorinated water; for high ammonia levels, perform partial water changes to reduce concentration, ensure that the filtration system is functioning properly, and consider increasing the frequency of water changes or enhancing the filtration system as needed
Step 9: Fish jumping from housing tanks or reaching humane endpoints	Tanks left uncovered; handling/transfer too abrupt; excessive agitation in the room; compromised health/insufficient acclimation; chronic poor water quality	If a fish jumps out of the housing tank, it should not be reintroduced into the experimental groups; additionally, if any fish reach a humane endpoint, they should be promptly removed from the tank and euthanized according to protocol
Steps 12, 14, 16, 28, 40, and 46: Fish jumping from stress tanks	Tanks not covered; fish startled during netting; prolonged air exposure; transfers performed too quickly/roughly	If a fish jumps out of the stress tank, it should not be reintroduced into the experimental groups; they should be promptly collected and euthanized according to protocol
Step 21: If any fish is hurt during the chasing protocol	Net frame contacting fish; overly aggressive chasing; overcrowded tank; inexperienced handler	If any fish sustain injuries during the chasing process, they should be assessed for humane endpoints; if necessary, they should be removed from the housing tank and euthanized according to protocol

### Understanding Results

**Figure 3:**
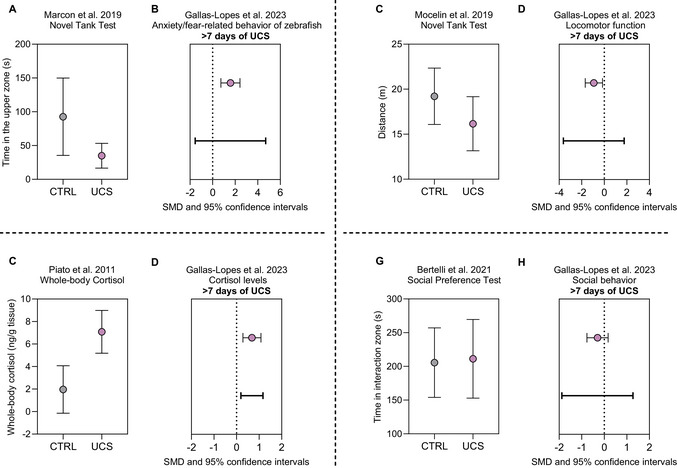
Illustration of various results obtained from studies implementing the UCS protocol in zebrafish, alongside meta‐analysis results of all studies published up to 2021. The data are presented as mean ± *SD* for experimental results and as standardized mean differences with 95% confidence intervals for meta‐analysis results. Bold lines indicate the prediction intervals. Panel (**A**) shows an example result for the time spent in the upper zone in a UCS study (Marcon et al., 2019). Panel (**B**) shows the meta‐analysis results depicting the standardized mean difference for the effects of UCS on anxiety/fear‐related behavior, along with the prediction interval (Gallas‐Lopes, Bastos, et al., 2023). Panel (**C**) shows an example result for the distance traveled in a UCS study (Mocelin et al., 2019). Panel (**D**) shows the meta‐analysis results depicting the standardized mean difference for the effects of UCS on locomotor function, along with the prediction interval (Gallas‐Lopes, Bastos, et al., 2023). Panel (**E**) shows an example result for whole body cortisol levels in a UCS study (Piato et al., 2011). Panel (**F**) shows the meta‐analysis results depicting the standardized mean difference for the effects of UCS on cortisol levels, along with the prediction interval (Gallas‐Lopes, Bastos, et al., 2023). Panel (**G**) shows an example result for the time spent in the interaction zone in a UCS study (Bertelli et al., 2021). Panel (**H**) shows the meta‐analysis results depicting the standardized mean difference for the effects of UCS on social behavior, along with the prediction interval (Gallas‐Lopes, Bastos, et al., 2023).

Based on our meta‐analysis of the effects of UCS in zebrafish, we anticipate several key outcomes from this protocol (Gallas‐Lopes, Bastos, et al., 2023). For experiments utilizing a UCS regimen of >7 days, it is expected to observe a significant increase in anxiety‐like behavior (Fig. 3A‐B), with a standardized mean difference (SMD) of 1.58 (95% CI: 0.73 to 2.43, *p* < .01) for the studies published up to 2021, with a prediction interval of –1.54 to 4.70. This finding is predominantly derived from studies employing the novel tank test, which assess the time spent in the upper zone as a primary indicator of the effects of stress. Significant differences in locomotor function are also anticipated between stressed and control groups (Fig. 3C‐D), with stressed animals exhibiting reduced mobility. Our meta‐analysis shows an SMD of −0.93 (95% CI: −1.69 to −0.16, *p* = .02) for the studies published up to 2021, with a prediction interval of –3.62 to 1.76. Locomotion is typically assessed in the novel tank test by measuring the total distance traveled, as reductions in distance and increased immobility may also be linked to anxiety‐like behavior. UCS protocols extending beyond 7 days are expected to result in a significant increase in cortisol levels (Fig. 3E‐F), with an SMD of 0.68 (95% CI: 0.28 to 1.08, *p* < .01) for the studies published up to 2021, with a prediction interval of 0.19 to 1.17. This reflects heightened stress responses at the biochemical level. Conversely, our analysis did not show significant effects of the UCS protocol on social behavior (SMD −0.30, 95% CI: −0.77 to 0.17, *p* = .1849), with a prediction interval of –1.88 to 1.27 (Fig. 3G‐H). This may suggest that social behavior in zebrafish is robust against even severe stress regimens. However, the limited number of studies assessing this outcome and the heterogeneity in results and methods must be considered. Additionally, it is important to account for publication bias. Egger's regression test indicated publication bias for most domains tested in our meta‐analysis: anxiety/fear‐related behavior (*p* = .001), locomotor function (*p* = .0277), and cortisol levels (*p* = .0566). All tests suggested a possible overestimation of the effects of UCS based on published data. For social behavior, the regression test was not statistically significant (*p* = .2507), but this result should be interpreted with caution due to the small number of studies reporting this outcome (Gallas‐Lopes, Bastos, et al., 2023). This analysis highlights the importance of publishing results regardless of the observed effect.

Eventually, if the UCS intervention is tested for its effect on a specific outcome for which little or no prior evidence exists, or that has not yet been validated in this model, the result might be negative. Although reasons for a negative result can be speculated, confirmation requires testing. Therefore, to validate the experiment, it is recommended to include an outcome that is known to be affected by this protocol, such as assessing time in the upper zone in the novel tank test, which has consistently been shown to be a reliable outcome, or measuring cortisol levels, as the prediction interval from the meta‐analysis suggests that most experiments should show higher cortisol levels in the stressed group.

### Time Considerations

Sample size estimation takes ∼30 min. Preparing housing tanks takes ∼2 hr for 4 tanks. Stress session 1 (tank change) takes ∼1 hr. Stress session 2 (chasing with a net) takes ∼20 min. Stress session 3 (overcrowding) takes ∼1.5 hr. Stress session 4 (low water level) takes ∼20 min. Stress session 5 (low water level) takes ∼20 min. Stress session 6 (cooling) takes ∼50 min. Stress session 7 (heating) takes ∼50 min. For stress sessions 8 to 28, timing varies according to stressor. For optional behavioral assessments, timing will depend on the test and the number of animals. Euthanasia and collection of samples take ∼10 min per fish. For optional biochemical/molecular assessments, timing will depend on the assay and the number of animals.

### Author Contributions

**Matheus Gallas‐Lopes**: Conceptualization; data curation; methodology; visualization; writing—original draft. **Ana Herrmann**: Conceptualization; funding acquisition; methodology; resources; supervision; writing—review and editing. **Angelo Piato**: Conceptualization; funding acquisition; methodology; resources; supervision; writing—review and editing.

### Conflict of Interest

The authors declare no conflicts of interest. The funders had no role in the study design, manuscript preparation, or the decision to submit the article for publication.

## Data Availability

Data sharing is not applicable to this article as no new data were created or analyzed in this study.

## References

[cpz170376-bib-0001] Aleström, P. , D'Angelo, L. , Midtlyng, P. J. , Schorderet, D. F. , Schulte‐Merker, S. , Sohm, F. , & Warner, S. (2020). Zebrafish: Housing and husbandry recommendations. Laboratory Animals, 54(3), 213–224. 10.1177/0023677219869037 31510859 PMC7301644

[cpz170376-bib-0002] Al‐Zoubi, R. M. , Abu‐Hijleh, H. , Zarour, A. , Zakaria, Z. Z. , Yassin, A. , Al‐Ansari, A. A. , Al‐Asmakh, M. , & Bawadi, H. (2024). Zebrafish model in illuminating the complexities of post‐traumatic stress disorders: A unique research tool. International Journal of Molecular Sciences, 25(9), 4895. 10.3390/ijms25094895 38732113 PMC11084870

[cpz170376-bib-0003] Atrooz, F. , Alkadhi, K. A. , & Salim, S. (2021). Understanding stress: Insights from rodent models. Current Research in Neurobiology, 2, 100013. 10.1016/j.crneur.2021.100013 36246514 PMC9559100

[cpz170376-bib-0004] Barcellos, L. J. G. , Ritter, F. , Kreutz, L. C. , Quevedo, R. M. , da Silva, L. B. , Bedin, A. C. , Finco, J. , & Cericato, L. (2007). Whole‐body cortisol increases after direct and visual contact with a predator in zebrafish, *Danio rerio* . Aquaculture, 272(1), 774–778. 10.1016/j.aquaculture.2007.09.002

[cpz170376-bib-0005] Barron, H. C. , Mars, R. B. , Dupret, D. , Lerch, J. P. , & Sampaio‐Baptista, C. (2020). Cross‐species neuroscience: Closing the explanatory gap. Philosophical Transactions of the Royal Society B: Biological Sciences, 376(1815), 20190633. 10.1098/rstb.2019.0633 PMC7116399 33190601

[cpz170376-bib-0006] Benneh, C. K. , Biney, R. P. , Mante, P. K. , Tandoh, A. , Adongo, D. W. , & Woode, E. (2017). Maerua angolensis stem bark extract reverses anxiety and related behaviours in zebrafish‐Involvement of GABAergic and 5‐HT systems. Journal of Ethnopharmacology, 207, 129–145. 10.1016/j.jep.2017.06.012 28645783

[cpz170376-bib-0007] Berg, I. , Härvelid, P. , Zürrer, W. E. , Rosso, M. , Reich, D. S. , & Ineichen, B. V. (2024). Which experimental factors govern successful animal‐to‐human translation in multiple sclerosis drug development? A systematic review and meta‐analysis. eBioMedicine, 110, 105434. 10.1016/j.ebiom.2024.105434 39515028 PMC11582441

[cpz170376-bib-0008] Bertelli, P. R. , Mocelin, R. , Marcon, M. , Sachett, A. , Gomez, R. , Rosa, A. R. , Herrmann, A. P. , & Piato, A. (2021). Anti‐stress effects of the glucagon‐like peptide‐1 receptor agonist liraglutide in zebrafish. Progress in Neuro‐Psychopharmacology and Biological Psychiatry, 111, 110388. 10.1016/j.pnpbp.2021.110388 34147534

[cpz170376-bib-0009] Buske, C. , & Gerlai, R. (2011). Shoaling develops with age in Zebrafish (*Danio rerio*). Progress in Neuro‐Psychopharmacology and Biological Psychiatry, 35(6), 1409–1415. 10.1016/j.pnpbp.2010.09.003 20837077 PMC3021101

[cpz170376-bib-0010] Cachat, J. , Stewart, A. , Grossman, L. , Gaikwad, S. , Kadri, F. , Chung, K. M. , Wu, N. , Wong, K. , Roy, S. , Suciu, C. , Goodspeed, J. , Elegante, M. , Bartels, B. , Elkhayat, S. , Tien, D. , Tan, J. , Denmark, A. , Gilder, T. , Kyzar, E. , … Kalueff, A. V. (2010). Measuring behavioral and endocrine responses to novelty stress in adult zebrafish. Nature Protocols, 5(11), 1786–1799. 10.1038/nprot.2010.140 21030954

[cpz170376-bib-0011] Chakravarty, S. , Reddy, B. R. , Sudhakar, S. R. , Saxena, S. , Das, T. , Meghah, V. , Brahmendra Swamy, C. V. , Kumar, A. , & Idris, M. M. (2013). Chronic Unpredictable Stress (CUS)‐induced anxiety and related mood disorders in a zebrafish model: Altered brain proteome profile implicates mitochondrial dysfunction. PLoS ONE, 8(5), e63302. 10.1371/journal.pone.0063302 23691016 PMC3653931

[cpz170376-bib-0012] Champagne, D. L. , Hoefnagels, C. C. M. , de Kloet, R. E. , & Richardson, M. K. (2010). Translating rodent behavioral repertoire to zebrafish (*Danio rerio*): Relevance for stress research. Behavioural Brain Research, 214(2), 332–342. 10.1016/j.bbr.2010.06.001 20540966

[cpz170376-bib-0013] Costa de Melo, N. , Sánchez‐Ortiz, B. L. , dos Santos Sampaio, T. I. , Matias Pereira, A. C. , Pinheiro da Silva Neto, F. L. , Ribeiro da Silva, H. , Alves Soares Cruz, R. , Keita, H. , Soares Pereira, A. M. , & Tavares Carvalho, J. C. (2019). Anxiolytic and antidepressant effects of the hydroethanolic extract from the leaves of aloysia polystachya (Griseb.) moldenke: A study on zebrafish (Danio rerio). Pharmaceuticals (Basel, Switzerland), 12(3), 106. 10.3390/ph12030106 31373315 PMC6789669

[cpz170376-bib-0014] de Abreu, M. S. , Costa, F. , Giacomini, A. C. V. V. , Demin, K. A. , Zabegalov, K. N. , Maslov, G. O. , Kositsyn, Y. M. , Petersen, E. V. , Strekalova, T. , Rosemberg, D. B. , & Kalueff, A. V. (2022). Towards modeling anhedonia and its treatment in Zebrafish. The International Journal of Neuropsychopharmacology, 25(4), 293–306. 10.1093/ijnp/pyab092 34918075 PMC9017771

[cpz170376-bib-0015] de Abreu, M. S. , Demin, K. A. , Giacomini, A. C. V. V. , Amstislavskaya, T. G. , Strekalova, T. , Maslov, G. O. , Kositsin, Y. , Petersen, E. V. , & Kalueff, A. V. (2021). Understanding how stress responses and stress‐related behaviors have evolved in zebrafish and mammals. Neurobiology of Stress, 15, 100405. 10.1016/j.ynstr.2021.100405 34722834 PMC8536782

[cpz170376-bib-0016] de Abreu, M. S. , Friend, A. J. , Demin, K. A. , Amstislavskaya, T. G. , Bao, W. , & Kalueff, A. V. (2018). Zebrafish models: Do we have valid paradigms for depression? Journal of Pharmacological and Toxicological Methods, 94(Pt 2), 16–22. 10.1016/j.vascn.2018.07.002 30030185

[cpz170376-bib-0017] Demers, N. E. , & Bayne, C. J. (1997). The immediate effects of stress on hormones and plasma lysozyme in rainbow trout. Developmental & Comparative Immunology, 21(4), 363–373. 10.1016/S0145-305X(97)00009-8 9303274

[cpz170376-bib-0018] Demin, K. A. , Lakstygal, A. M. , Krotova, N. A. , Masharsky, A. , Tagawa, N. , Chernysh, M. V. , Ilyin, N. P. , Taranov, A. S. , Galstyan, D. S. , Derzhavina, K. A. , Levchenko, N. A. , Kolesnikova, T. O. , Mor, M. S. , Vasyutina, M. L. , Efimova, E. V. , Katolikova, N. , Prjibelski, A. D. , Gainetdinov, R. R. , de Abreu, M. S. , … Kalueff, A. V. (2020). Understanding complex dynamics of behavioral, neurochemical and transcriptomic changes induced by prolonged chronic unpredictable stress in zebrafish. Scientific Reports, 10(1), 19981. 10.1038/s41598-020-75855-3 33203921 PMC7673038

[cpz170376-bib-0019] dos Santos Sampaio, T. I. , de Melo, N. C. , de Freitas Paiva, B. T. , da Silva Aleluia, G. A. , da Silva Neto, F. L. P. , da Silva, H. R. , Keita, H. , Cruz, R. A. S. , Sánchez‐Ortiz, B. L. , Pineda‐Peña, E. A. , Balderas, J. L. , Navarrete, A. , & Carvalho, J. C. T. (2018). Leaves of Spondias mombin L. a traditional anxiolytic and antidepressant: Pharmacological evaluation on zebrafish (Danio rerio). Journal of Ethnopharmacology, 224, 563–578. 10.1016/j.jep.2018.05.037 29852265

[cpz170376-bib-0020] Egan, R. J. , Bergner, C. L. , Hart, P. C. , Cachat, J. M. , Canavello, P. R. , Elegante, M. F. , Elkhayat, S. I. , Bartels, B. K. , Tien, A. K. , Tien, D. H. , Mohnot, S. , Beeson, E. , Glasgow, E. , Amri, H. , Zukowska, Z. , & Kalueff, A. V. (2009). Understanding behavioral and physiological phenotypes of stress and anxiety in zebrafish. Behavioural Brain Research, 205(1), 38–44. 10.1016/j.bbr.2009.06.022 19540270 PMC2922906

[cpz170376-bib-0021] Fulcher, N. , Tran, S. , Shams, S. , Chatterjee, D. , & Gerlai, R. (2017). Neurochemical and behavioral responses to unpredictable chronic mild stress following developmental isolation: The zebrafish as a model for major depression. Zebrafish, 14(1), 23–34. 10.1089/zeb.2016.1295 27454937

[cpz170376-bib-0022] Gallas‐Lopes, M. , Bastos, L. M. , Benvenutti, R. , Panzenhagen, A. C. , Piato, A. , & Herrmann, A. P. (2023). Systematic review and meta‐analysis of 10 years of unpredictable chronic stress in zebrafish. Lab Animal, 52(10), 229–246. 10.1038/s41684-023-01239-5 37709998

[cpz170376-bib-0023] Gallas‐Lopes, M. , Benvenutti, R. , Donzelli, N. I. Z. , & Marcon, M. (2023). A systematic review of the impact of environmental enrichment in zebrafish. Lab Animal, 52(12), 332–343. 10.1038/s41684-023-01288-w 38017181

[cpz170376-bib-0024] Gerlai, R. (2010a). High‐throughput behavioral screens: The first step towards finding genes involved in vertebrate brain function using zebrafish. Molecules, 15(4), 2609–2622. 10.3390/molecules15042609 20428068 PMC6257226

[cpz170376-bib-0025] Gerlai, R. (2010b). Zebrafish antipredatory responses: A future for translational research? Behavioural Brain Research, 207(2), 223–231. 10.1016/j.bbr.2009.10.008 19836422 PMC3203216

[cpz170376-bib-0026] Gorelick, D. A. , & Habenicht, L. M. (2020). Chapter 17—Endocrine Systems. In S. C. Cartner , J. S. Eisen , S. C. Farmer , K. J. Guillemin , M. L. Kent , & G. E. Sanders (Eds.), The Zebrafish in Biomedical Research (pp. 165–179). Academic Press. 10.1016/B978-0-12-812431-4.00017-8

[cpz170376-bib-0027] Grzelak, A. K. , Davis, D. J. , Caraker, S. M. , Crim, M. J. , Spitsbergen, J. M. , & Wiedmeyer, C. E. (2017). Stress leukogram induced by acute and chronic stress in zebrafish (Danio rerio). Comparative Medicine, 67(3), 263–269. 28662755 PMC5482518

[cpz170376-bib-0028] Hooijmans, C. R. , Rovers, M. M. , de Vries, R. B. , Leenaars, M. , Ritskes‐Hoitinga, M. , & Langendam, M. W. (2014). SYRCLE's risk of bias tool for animal studies. BMC Medical Research Methodology, 14(1), 43. 10.1186/1471-2288-14-43 24667063 PMC4230647

[cpz170376-bib-0029] Katz, R. J. (1982). Animal model of depression: Pharmacological sensitivity of a hedonic deficit. Pharmacology, Biochemistry, and Behavior, 16(6), 965–968. 10.1016/0091-3057(82)90053-3 7202217

[cpz170376-bib-0030] Katz, R. J. , & Hersh, S. (1981). Amitriptyline and scopolamine in an animal model of depression. Neuroscience and Biobehavioral Reviews, 5(2), 265–271. 10.1016/0149-7634(81)90008-7 7196557

[cpz170376-bib-0031] Katz, R. J. , Roth, K. A. , & Carroll, B. J. (1981). Acute and chronic stress effects on open field activity in the rat: Implications for a model of depression. Neuroscience and Biobehavioral Reviews, 5(2), 247–251. 10.1016/0149-7634(81)90005-1 7196554

[cpz170376-bib-0032] Kirsten, K. , Pompermaier, A. , Koakoski, G. , Mendonça‐Soares, S. , da Costa, R. A. , Maffi, V. C. , Kreutz, L. C. , & Barcellos, L. J. G. (2021). Acute and chronic stress differently alter the expression of cytokine and neuronal markers genes in zebrafish brain. Stress (Amsterdam, Netherlands), 24(1), 107–112. 10.1080/10253890.2020.1724947 32013653

[cpz170376-bib-0033] Kütter, M. T. , Barcellos, L. J. G. , Boyle, R. T. , Marins, L. F. , & Silveira, T. (2023). Good practices in the rearing and maintenance of zebrafish (*Danio rerio*) in Brazilian laboratories. Ciência Animal Brasileira, 24, e. 10.1590/1809-6891v24e-74134E

[cpz170376-bib-0034] Kysil, E. V. , Meshalkina, D. A. , Frick, E. E. , Echevarria, D. J. , Rosemberg, D. B. , Maximino, C. , Lima, M. G. , Abreu, M. S. , Giacomini, A. C. , Barcellos, L. J. G. , Song, C. , & Kalueff, A. V. (2017). Comparative analyses of zebrafish anxiety‐like behavior using conflict‐based novelty tests. Zebrafish, 14(3), 197–208. 10.1089/zeb.2016.1415 28459655

[cpz170376-bib-0035] Manuel, R. , Gorissen, M. , Zethof, J. , Ebbesson, L. O. E. , van de Vis, H. , Flik, G. , & van den Bos, R. (2014). Unpredictable chronic stress decreases inhibitory avoidance learning in Tuebingen long‐fin zebrafish: Stronger effects in the resting phase than in the active phase. The Journal of Experimental Biology, 217(Pt 21), 3919–3928. 10.1242/jeb.109736 25267842

[cpz170376-bib-0036] Marcon, M. , Herrmann, A. P. , Mocelin, R. , Rambo, C. L. , Koakoski, G. , Abreu, M. S. , Conterato, G. M. , Kist, L. W. , Bogo, M. R. , Zanatta, L. , Barcellos, L. J. , & Piato, A. L. (2016). Prevention of unpredictable chronic stress‐related phenomena in zebrafish exposed to bromazepam, fluoxetine and nortriptyline. Psychopharmacology, 233(21–22), 3815–3824. 10.1007/s00213-016-4408-5 27562666

[cpz170376-bib-0037] Marcon, M. , Mocelin, R. , Benvenutti, R. , Costa, T. , Herrmann, A. P. , de Oliveira, D. L. , Koakoski, G. , Barcellos, L. J. G. , & Piato, A. (2018). Environmental enrichment modulates the response to chronic stress in zebrafish. The Journal of Experimental Biology, 221(Pt 4), jeb176735. 10.1242/jeb.176735 29361609

[cpz170376-bib-0038] Marcon, M. , Mocelin, R. , de Oliveira, D. L. , da Rosa Araujo, A. S. , Herrmann, A. P. , & Piato, A. (2019). Acetyl‐L‐carnitine as a putative candidate for the treatment of stress‐related psychiatric disorders: Novel evidence from a zebrafish model. Neuropharmacology, 150, 145–152. 10.1016/j.neuropharm.2019.03.024 30917915

[cpz170376-bib-0039] Marcon, M. , Mocelin, R. , Sachett, A. , Siebel, A. M. , Herrmann, A. P. , & Piato, A. (2018). Enriched environment prevents oxidative stress in zebrafish submitted to unpredictable chronic stress. PeerJ, 6, e5136. 10.7717/peerj.5136 30002970 PMC6035866

[cpz170376-bib-0040] Marx, W. , Penninx, B. W. J. H. , Solmi, M. , Furukawa, T. A. , Firth, J. , Carvalho, A. F. , & Berk, M. (2023). Major depressive disorder. Nature Reviews Disease Primers, 9(1), 1–21. 10.1038/s41572-023-00454-1 37620370

[cpz170376-bib-0041] Maximino, C. , Marques de Brito, T. , Dias, C. A. G. D. M. , Gouveia, A. , & Morato, S. (2010). Scototaxis as anxiety‐like behavior in fish. Nature Protocols, 5(2), 209–216. 10.1038/nprot.2009.225 20134420

[cpz170376-bib-0042] Mocelin, R. , Marcon, M. , D'ambros, S. , Mattos, J. , Sachett, A. , Siebel, A. M. , Herrmann, A. P. , & Piato, A. (2019). N‐acetylcysteine reverses anxiety and oxidative damage induced by unpredictable chronic stress in zebrafish. Molecular Neurobiology, 56(2), 1188–1195. 10.1007/s12035-018-1165-y 29876880

[cpz170376-bib-0043] Neves, N. A. , Gallas‐Lopes, M. , Patelli‐Alves, A. , Müller, D. V. , Bastos, L. M. , Stahlhofer‐Buss, T. , Herrmann, A. P. , , & Piato, A. (2026). Unpredictable chronic stress fails to induce robust changes in zebrafish social behavior. Neuroscience Letters, 878, 138594. 10.1016/j.neulet.2026.138594 41903864

[cpz170376-bib-0044] Nguyen, M. , Stewart, A. M. , & Kalueff, A. V. (2014). Aquatic blues: Modeling depression and antidepressant action in zebrafish. Progress in Neuro‐Psychopharmacology and Biological Psychiatry, 55, 26–39. 10.1016/j.pnpbp.2014.03.003 24657522

[cpz170376-bib-0045] Pavlidis, M. , Theodoridi, A. , & Tsalafouta, A. (2015). Neuroendocrine regulation of the stress response in adult zebrafish, Danio rerio. Progress in Neuro‐Psychopharmacology & Biological Psychiatry, 60, 121–131. 10.1016/j.pnpbp.2015.02.014 25748166

[cpz170376-bib-0046] Percie du Sert, N. , Hurst, V. , Ahluwalia, A. , Alam, S. , Avey, M. T. , Baker, M. , Browne, W. J. , Clark, A. , Cuthill, I. C. , Dirnagl, U. , Emerson, M. , Garner, P. , Holgate, S. T. , Howells, D. W. , Karp, N. A. , Lazic, S. E. , Lidster, K. , MacCallum, C. J. , Macleod, M. , … Würbel, H. (2020). The ARRIVE guidelines 2.0: Updated guidelines for reporting animal research. PLoS Biology, 18(7), e3000410. 10.1371/journal.pbio.3000410 32663219 PMC7360023

[cpz170376-bib-0047] Piato, Â. L. , Capiotti, K. M. , Tamborski, A. R. , Oses, J. P. , Barcellos, L. J. G. , Bogo, M. R. , Lara, D. R. , Vianna, M. R. , & Bonan, C. D. (2011). Unpredictable chronic stress model in zebrafish (*Danio rerio*): Behavioral and physiological responses. Progress in Neuro‐Psychopharmacology and Biological Psychiatry, 35(2), 561–567. 10.1016/j.pnpbp.2010.12.018 21187119

[cpz170376-bib-0048] Quadros, V. A. , Rosa, L. V. , Costa, F. V. , Koakoski, G. , Barcellos, L. J. G. , & Rosemberg, D. B. (2021). Predictable chronic stress modulates behavioral and neuroendocrine phenotypes of zebrafish: Influence of two homotypic stressors on stress‐mediated responses. Comparative Biochemistry and Physiology Part C: Toxicology & Pharmacology, 247, 109030. 10.1016/j.cbpc.2021.109030 33722767

[cpz170376-bib-0049] Ramsay, J. M. , Feist, G. W. , Varga, Z. M. , Westerfield, M. , Kent, M. L. , & Schreck, C. B. (2006). Whole‐body cortisol is an indicator of crowding stress in adult zebrafish, *Danio rerio* . Aquaculture, 258(1), 565–574. 10.1016/j.aquaculture.2006.04.020

[cpz170376-bib-0050] Ramsay, J. M. , Feist, G. W. , Varga, Z. M. , Westerfield, M. , Kent, M. L. , & Schreck, C. B. (2009). Whole‐body cortisol response of zebrafish to acute net handling stress. Aquaculture (Amsterdam, Netherlands), 297(1–4), 157–162. 25587201 10.1016/j.aquaculture.2009.08.035 PMC4289633

[cpz170376-bib-0051] Reddy, B. R. , Babu, N. S. , Das, T. , Bhattacharya, D. , Murthy, C. L. N. , Kumar, A. , Idris, M. M. , & Chakravarty, S. (2021). Proteome profile of telencephalon associates attenuated neurogenesis with chronic stress induced mood disorder phenotypes in zebrafish model. Pharmacology, Biochemistry, and Behavior, 204, 173170. 10.1016/j.pbb.2021.173170 33684455

[cpz170376-bib-0052] Reddy, R. G. , Dachavaram, S. S. , Reddy, B. R. , Kalyankar, K. B. , Rajan, W. D. , Kootar, S. , Kumar, A. , Das, S. , & Chakravarty, S. (2018). Fellutamide B synthetic path intermediates with in vitro neuroactive function shows mood‐elevating effect in stress‐induced zebrafish model. ACS Omega, 3(9), 10534–10544. 10.1021/acsomega.8b00456 30320245 PMC6173481

[cpz170376-bib-0053] Reddy, R. G. , Surineni, G. , Bhattacharya, D. , Marvadi, S. K. , Sagar, A. , Kalle, A. M. , Kumar, A. , Kantevari, S. , & Chakravarty, S. (2019). Crafting carbazole‐based vorinostat and tubastatin‐A‐like Histone Deacetylase (HDAC) inhibitors with potent in vitro and in vivo neuroactive functions. ACS Omega, 4(17), 17279–17294. 10.1021/acsomega.9b01950 31656902 PMC6811854

[cpz170376-bib-0054] Rotllant, J. , & Tort, L. (1997). Cortisol and glucose responses after acute stress by net handling in the sparid red porgy previously subjected to crowding stress. Journal of Fish Biology, 51(1), 21–28. 10.1111/j.1095-8649.1997.tb02510.x 9236085

[cpz170376-bib-0055] Sachett, A. , Gallas‐Lopes, M. , Benvenutti, R. , Marcon, M. , Aguiar, G. P. S. , Herrmann, A. P. , Vladimir Oliveira, J. , Siebel, A. M. , & Piato, A. (2022). Curcumin micronization by supercritical fluid: In vitro and in vivo biological relevance. Industrial Crops and Products, 177, 114501. 10.1016/j.indcrop.2021.114501

[cpz170376-bib-0056] Serra, E. L. , Medalha, C. C. , & Mattioli, R. (1999). Natural preference of zebrafish (Danio rerio) for a dark environment. Brazilian Journal of Medical and Biological Research, 32, 1551–1553. 10.1590/S0100-879X1999001200016 10585639

[cpz170376-bib-0057] Smith, A. J. , Clutton, R. E. , Lilley, E. , Hansen, K. E. A. , & Brattelid, T. (2018). PREPARE: Guidelines for planning animal research and testing. Laboratory Animals, 52(2), 135–141. 10.1177/0023677217724823 28771074 PMC5862319

[cpz170376-bib-0058] Song, C. , Liu, B.‐P. , Zhang, Y.‐P. , Peng, Z. , Wang, J. , Collier, A. D. , Echevarria, D. J. , Savelieva, K. V. , Lawrence, R. F. , Rex, C. S. , Meshalkina, D. A. , & Kalueff, A. V. (2018). Modeling consequences of prolonged strong unpredictable stress in zebrafish: Complex effects on behavior and physiology. Progress in Neuro‐Psychopharmacology & Biological Psychiatry, 81, 384–394. 10.1016/j.pnpbp.2017.08.021 28847526

[cpz170376-bib-0059] Tran, I. , & Gellner, A.‐K. (2023). Long‐term effects of chronic stress models in adult mice. Journal of Neural Transmission, 130(9), 1133–1151. 10.1007/s00702-023-02598-6 36786896 PMC10460743

[cpz170376-bib-0060] Tran, S. , & Gerlai, R. (2016). The novel tank test: Handling stress and the context specific psychopharmacology of anxiety. Current Psychopharmacology, 5(2), 169–179. 10.2174/2211556005666160519144414

[cpz170376-bib-0061] Underwood, W. , & Anthony, R. (2020). AVMA guidelines for the euthanasia of animals: 2020 edition. Retrieved on March, 2013(30), 2020–1.

[cpz170376-bib-0062] Willner, P. (1997). Validity, reliability and utility of the chronic mild stress model of depression: A 10‐year review and evaluation. Psychopharmacology, 134(4), 319–329. 10.1007/s002130050456 9452163

[cpz170376-bib-0063] Willner, P. (2017). The chronic mild stress (CMS) model of depression: History, evaluation and usage. Neurobiology of Stress, 6, 78–93. 10.1016/j.ynstr.2016.08.002 28229111 PMC5314424

[cpz170376-bib-0064] Willner, P. , Towell, A. , Sampson, D. , Sophokleous, S. , & Muscat, R. (1987). Reduction of sucrose preference by chronic unpredictable mild stress, and its restoration by a tricyclic antidepressant. Psychopharmacology, 93(3), 358–364. 10.1007/BF00187257 3124165

[cpz170376-bib-0065] Zuccarelli, M. D. , & Ingermann, R. L. (2007). Exhaustive exercise, animal stress, and environmental hypercapnia on motility of sperm of steelhead trout (*Oncorhynchus mykiss*). Comparative Biochemistry and Physiology Part A: Molecular & Integrative Physiology, 147(1), 247–253. 10.1016/j.cbpa.2006.12.040 17303460

[cpz170376-bib-0067] Piato et al. (2011). See above.

[cpz170376-bib-0068] *This study presents the first adaptation of the unpredictable chronic stress (UCS) protocol for adult zebrafish, describing the stressor set and demonstrating behavioral and endocrine stress‐related outcomes*.

[cpz170376-bib-0069] Gallas‐Lopes, Bastos, et al. (2023). See above.

[cpz170376-bib-0070] *This systematic review and meta‐analysis summarize ten years of UCS research in zebrafish, identifying consistent behavioral and biochemical effects, sources of heterogeneity, and methodological limitations that support the need for protocol standardization*.

